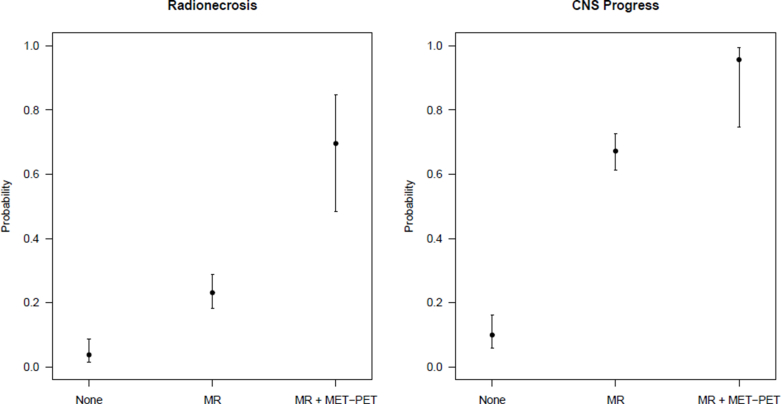# Irradiated tumor volume as a predictor of local recurrence and radionecrosis in lung cancer with brain metastases treated with stereotactic radiosurgery

**DOI:** 10.1016/j.esmoop.2025.106028

**Published:** 2026-01-02

**Authors:** A. Koulouris, O. Grundberg, M. Skribek, C. Kamali, P. Hydbring, M. Gubanski, S. Ekman, G. Tsakonas

**Affiliations:** 1 Thoracic Oncology Center, Karolinska University Hospital, Stockholm; 2 Department of Oncology-Pathology, Karolinska Institutet, Stockholm, Sweden; 3 Laboratory of Translational Oncology, Medical School, University of Crete, Heraklion, Greece; 4 Department of Molecular Medicine and Surgery, Karolinska Institutet, Stockholm; 5 Department of Radiotherapy, Karolinska University Hospital, Stockholm, Sweden

**Keywords:** lung cancer, brain metastases, stereotactic radiosurgery, radionecrosis, local recurrence

## Abstract

**Background:**

Stereotactic radiosurgery (SRS) is a standard treatment of brain metastases (BM), but it may lead to radionecrosis (RN) or local recurrence (LR). This study evaluates irradiated tumor volume as a predictor of LR and RN in SRS-treated lung cancer patients with BM.

**Methods:**

We retrospectively analyzed 431 lung cancer patients with BM who underwent SRS at Karolinska University Hospital (all-comers, 2009-2020). Associations among irradiated tumor volume and risks of RN, symptomatic RN, and LR at 6 and 12 months were assessed using Cox regression models. Furthermore, we evaluated the diagnostic performance of methionine positron emission tomography–computed tomography (PET-CT) in differentiating RN from LR.

**Results:**

Forty patients (9.3%) developed asymptomatic RN, 36 (8.4%) symptomatic RN, and 67 (15.5%) LR. Larger tumor volumes significantly increased RN and LR risks. At 6 months, a volume of 4.75 cm^3^ corresponded to an RN risk reaching up to 20%. By 12 months, substantially smaller volumes were correlated with the same risk levels. Symptomatic RN followed a similar trend, at 6 months and 12 months. A 20% risk of LR was observed with volumes of 5.66 cm^3^ and 3.28 cm^3^ at 6 months and 12 months, respectively. Methionine PET-CT had a sensitivity of 0.909 and specificity of 0.600 in detecting RN or LR, with no significant diagnostic advantage compared with magnetic resonance imaging (MRI).

**Conclusion:**

Larger irradiated tumor volumes were correlated with increased RN or LR risk. Smaller volumes can lead to RN and LR, not evident at 6 months, but emerging by 12 months after SRS. Methionine PET-CT showed high sensitivity but modest specificity and offered no significant benefit over MRI in differentiating RN from LR.

## Highlights

• Brain metastases from lung cancer face risks of RN and recurrence after SRS.
• Irradiated tumor volume predicts RN and local failure in SRS-treated CNS metastases.
• Small volumes can cause delayed RN and LR, seen by 12 months after SRS.
• Methionine PET-CT showed no clear advantage over MRI in differentiating local recurrence from RN.

## Graphical abstract

**Figure:**
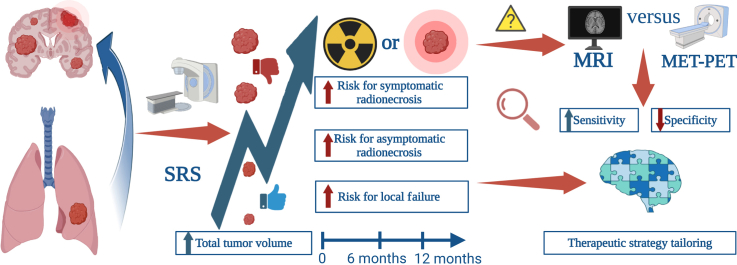

CNS metastases volume: a predictor for recurrence and radionecrosis. Created in BioRender. Ekman, S. (2025) https://BioRender.com/c15s098. CNS, central nervous system; MET-PET, methionine positron emission tomography–computed tomography; MRI, magnetic resonance imaging; SRS, stereotactic radiosurgery.

## Introduction

The cumulative incidence of brain metastases (BM) in non-small-cell lung cancer (NSCLC) patients is estimated to range from 20% to 50%, representing a significant negative prognostic factor.^1^, ^2^, ^3^, ^4^, ^5^ With the advent of targeted therapies and immune checkpoint inhibitors (ICI), intracranial control and overall survival (OS) have improved substantially, making the prevention and recognition of late complications increasingly important.^6^, ^7^, ^8^, ^9^

Stereotactic radiosurgery (SRS) constitutes an effective treatment modality, particularly for younger patients with a limited number of BM, well-controlled extracranial disease, and favorable Eastern Cooperative Oncology Group performance status (ECOG-PS).^10^ SRS can be effectively and safely complemented by systemic treatment, yielding significant intracranial responses, yet most prior studies have evaluated it in heterogeneous BM cohorts from multiple primaries, limiting disease-specific insights.^8^^,^^11^, ^12^, ^13^, ^14^, ^15^ Importantly, BM represent a heterogeneous entity, with survival outcomes and risk factors strongly influenced by the underlying primary malignancy, underscoring the need for diagnosis-specific analyses.^16^

Beyond clinical heterogeneity, growing evidence points to distinct immunobiology in BM from NSCLC compared with other malignancies. The contraction of T-cell clones within BM, despite a higher mutational burden compared with paired primaries, suggests an immune-restricted microenvironment that may influence recurrence and treatment-related toxicity when SRS is combined with systemic therapies.^17^ Additional molecular mechanisms contribute, including epithelial–mesenchymal transition (EMT) with E-cadherin loss and MMP up-regulation, as well as Nrf2+/CK+ cell density and dysregulated microRNAs (e.g. miR-219, miR-199), all linked to central nervous system (CNS) relapse and metastatic progression.^18^, ^19^, ^20^ Collectively, these findings highlight the biological distinctiveness of NSCLC patients with BM and the need for tumor-specific risk stratification.

The need for disease-specific analyses is reflected clinically by NSCLC-specific prognostic indices such as Diagnosis-Specific Grade Prognostic Assessment (DS-GPA), Lung-molGPA, and SRS Brain Prognostic Index (SRS-BPI), which incorporate histology, genetic alterations, and extracranial disease status as prognostic biomarkers.^21^, ^22^, ^23^ Moreover, patients receiving targeted therapy or ICI may show unique intracranial relapse patterns and toxicity profiles, reinforcing the need for dedicated analyses in this patient population.^7^^,^^9^^,^^16^

In prognostic models and guidelines, tumor volume has emerged as a major prognostic factor, with larger cumulative volumes linked to poorer outcomes and higher complication rates.^24^, ^25^, ^26^, ^27^ According to the European Association of Neuro-oncology (EANO)–European Society for Medical Oncology (ESMO) guidelines, SRS should be avoided for total BM volumes exceeding 15 cm^3^.^1^^,^^24^ Local recurrence (LR) and radionecrosis (RN) are recognized post-SRS complications, with LR occurring in ∼20% of larger metastases within 12 months.^28^ Risk factors for LR include tumor size, dose, and the number of irradiated lesions, while RN is associated with high radiation doses, larger treated volumes, and systemic therapies, such as immunotherapy.^27^^,^^29^, ^30^, ^31^

Differentiating LR from RN remains a clinical challenge due to overlapping radiographic features, even with advanced magnetic resonance imaging (MRI) techniques.^32^ Functional imaging modalities, such as ^11^C-methionine positron emission tomography–computed tomography (PET-CT), have demonstrated potential in distinguishing between the two conditions with high sensitivity and specificity.^33^^,^^34^ Despite encouraging findings, these methods have primarily been validated in mixed BM populations, leaving a gap in understanding their utility in lung cancer-specific cohorts.

This study aims to evaluate irradiated tumor volume as a risk factor for LR and RN in a large cohort of lung cancer patients treated with SRS. Furthermore, it explores the diagnostic performance of ^11^C-methionine PET-CT in differentiating LR from RN in a real-world clinical setting, contributing to improved management of post-SRS complications in this high-risk population.

## Patients and methods

### Study population and data collection

**Figure 1:**
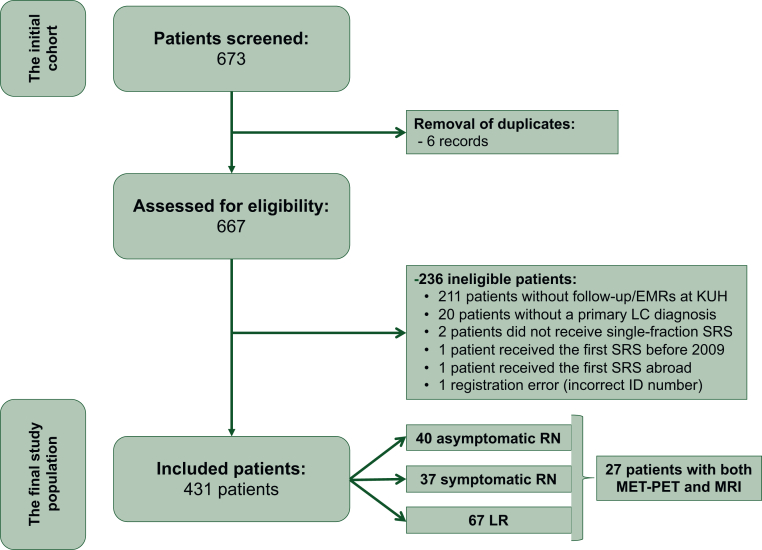
**Flow diagram of the study.** EMRs, electronic medical records; KUH, Karolinska University Hospital; LC, lung cancer; LR, local recurrence; MET-PET, methionine positron emission tomography–computed tomography; RN, radionecrosis; SRS, stereotactic radiosurgery.

A retrospective analysis was carried out on a cohort of 673 patients with CNS metastases who underwent SRS using Gamma Knife between December 2009 and September 2020 at Karolinska University Hospital (KUH) in Stockholm, Sweden. The cohort was identified through KUH’s electronic medical records (EMR). As KUH is the sole provider of Gamma Knife in Sweden and the only regional center for lung cancer care in Stockholm, this cohort represents a comprehensive all-comers population from the Stockholm area. All SRS treatments were delivered in a single fraction using the Leksell Gamma Knife Perfexion (Elekta, Stockholm, Sweden) with frame fixation. The marginal prescribed dose ranged from 18 Gy to 25 Gy, depending on the size of the lesion. Following the exclusion of duplicates, patients without a primary lung cancer diagnosis, and those referred from outside the region, the final study population included 431 patients (Figure 1).

Demographic, clinical, and radiological data were extracted retrospectively from EMR and radiological charts. The dataset included patient age, Eastern Cooperative Oncology Group performance status (ECOG-PS), smoking history, tumor histology, genetic alterations, programmed death-ligand 1 (PD-L1) expression, metastatic disease burden, number and sites of metastases, and detailed information on BM characteristics. Molecular profiling was carried out for all lung cancer patients during the study period at KUH using either next-generation sequencing (NGS) or an RT–PCR-based targeted panel, irrespective of histological subtype. Additional information encompassed the presence of neurological symptoms, the date of BM diagnosis, and the date of the first SRS application, as well as a comprehensive review of systemic and local treatments for BM, including angiogenesis inhibitors. Radiological follow-up data included the imaging modality used and the diagnosis of LR or RN. Outcome measures, including OS, were also recorded (Supplementary Table S1, available at https://doi.org/10.1016/j.esmoop.2025.106028).

Informed consent was obtained from all patients who were alive at the time of data collection. The study received approval from the regional ethical review board (Dnr 2021-05428-01) and adhered to the principles outlined in the Declaration of Helsinki.

### Volume assessment

The volume of treated BM was determined through a detailed review of stereotactic MRI examinations. In particular, measurements included the number of all BM, the diameter and volume of the largest brain metastatic lesion, as well as the total volume of all treated BM. For patients undergoing multiple SRS treatments, only the volumes from the initial SRS session were recorded.

### Imaging methods

Post-SRS imaging follow-up was conducted using either contrast-enhanced CT, MRI, or ^11^C-methionine PET-CT. In accordance with clinical practice, follow-up MRI was scheduled approximately every 3 months when feasible, with intervals individualized based on systemic treatment response, extracranial disease status, and any new or evolving neurologic symptoms. All MRI scans were conducted at the Neuroradiology Unit of KUH and reviewed by experienced neuroradiologists. For patients unable to undergo MRI due to logistical constraints, contraindications, or declining PS, contrast-enhanced CT scans were carried out as an alternative. For analysis, patients were classified as (i) MRI-based follow-up, (ii) MRI plus methionine PET-CT, and (iii) no-MRI follow-up, which included CT-only and no post-SRS imaging.

Methionine PET was utilized in cases where either LR or RN was suspected, typically following evidence of progression on MRI. It was primarily reserved for cases in which MRI, including perfusion sequences when available, did not allow a confident distinction between the two. When both MRI and PET-CT were carried out, classification was based on multidisciplinary consensus integrating imaging and clinical course, with histopathology superseding imaging when available. All methionine PET-CT scans were carried out at the Nuclear Medicine Unit of KUH and interpreted by trained nuclear medicine specialists and neuroradiologists. LR and RN were differentiated using the lesion-to-normal ratio (LNR) of standardized uptake values (SUV). In this study, an LNR cut-off of >2 was employed, based on findings by Kits et al., in which an SUV_max_ mirror threshold of ≥1.99 demonstrated 100% specificity for identifying tumor recurrence in similar clinical scenarios.^35^

### Definitions and management of suspected LR and RN

LR was defined as the presence of one or more of the following criteria: histopathologic confirmation of recurrence following tumor resection or biopsy, death attributable to progression of BM, continuous enlargement of the lesion observed on serial MRI, or a strong suspicion of recurrence by the nuclear physician, supported by lesion shrinkage after systemic or local therapy. In parallel, RN was identified based on either histopathologic confirmation following biopsy or resection, or radiological evidence of lesion stabilization or progression of necrotic changes without significant increase in volume or the appearance of new CNS lesions during follow-up imaging.

Imaging features favoring RN included heterogeneous or ring-like enhancement with a central hypointense component on T1 after contrast and prominent T2/fluid-attenuated inversion recovery (FLAIR) edema, stability or spontaneous decrease of the enhancing component on two or more interval MRIs without new systemic treatment, and qualitatively low perfusion on perfusion MRI (when available). In contrast, LR was characterized by progressive enlargement of the enhancing component on serial MRI, nodular or thick irregular enhancement at the SRS margin, and elevated perfusion on perfusion MRI relative to contralateral white matter. These criteria are consistent with the Response Assessment in Neuro-Oncology Brain Metastases (RANO-BM) recommendations.^36^

cases of LR and RN were reviewed by a multidisciplinary team comprising neuroradiologists, neuro-oncologists, and neurosurgeons. Diagnostic evaluations incorporated MRI findings, methionine PET-CT uptake patterns, lesion location, progression of non-SRS-treated lesions, systemic disease status, and prior treatment history. Histopathology, when available, was considered the definitive standard and overruled imaging-based classifications. CT-only examinations were not considered sufficient to adjudicate RN versus LR. Patients were further classified as symptomatic or asymptomatic based on clinical presentation. Symptomatic cases were characterized by either focal neurological deficits or generalized symptoms, such as headache, nausea, focal or generalized seizures, and cognitive impairment.

### Statistical analysis

The occurrence of RN, symptomatic RN, and LR was assessed according to the aforementioned definitions. Descriptive statistics were used to summarize the patient population and subgroups. OS was measured in months from the date of SRS to either the last follow-up or death, with 10 January 2023 as the censoring date. For RN and LR analyses, patients were censored at the time of death or last available follow-up if no event had occurred. Patients with missing imaging follow-up were excluded from modality-specific analyses. Associations between total irradiated tumor volume and the risks of RN, symptomatic RN, and LR at 6 and 12 months were analyzed using Cox regression models. Tumor volume was modeled with penalized splines to account for potential nonlinear relationships. The risk predictions at 6 months and 12 months were plotted against tumor volume, with corresponding 95% confidence intervals (CIs). Moreover, calibration curves for predicting radionecrosis and CNS progression were generated using the Efron–Gong bootstrap method to assess model performance.

To evaluate the diagnostic performance of methionine PET-CT in distinguishing RN from LR, diagnoses based on MRI were used as the reference standard. Sensitivity and specificity were calculated using 2 × 2 tables, with additional analyses conducted using kappa statistics and McNemar’s test to evaluate agreement and diagnostic differences. The use of MRI, both MRI and methionine PET, or neither was used as an independent variable in a logistic regression model in order to calculate the probability of RN or CNS progression.

All statistical analyses were conducted using R software version 4.2.2, utilizing the glmnet, polspline, boot, survival, Epi, and rms add-on packages (R Foundation for Statistical Computing, Vienna, Austria; available at https://www.r-project.org/).

## Results

### Patient characteristics

A total of 431 lung cancer patients with BM treated with SRS were included in the analysis. The median follow-up time, defined as the interval from first SRS to last follow-up or death, was 8 months, with an interquartile range (IQR) of 3-19 months. Notably, 25 patients had follow-up exceeding 5 years, including 6 followed for >10 years. Using the specified criteria for defining LR and RN, we identified 40 patients with asymptomatic RN, 36 with symptomatic RN, and 67 with LR (Figure 1). Descriptive statistics for the entire cohort, as well as for the subgroups of patients with asymptomatic RN, symptomatic RN, and LR, are summarized in Table 1. At the time of BM diagnosis, 140 patients (32.5%) had a single lesion, 144 (33.4%) had two or three BM, and 147 (34.1%) presented with four or more lesions. It is worth mentioning that 15 patients in the cohort had cumulative tumor volumes exceeding the conventional threshold of 15 cm^3^. Among the 76 patients diagnosed with RN, 71 (93.4%) developed RN in a lesion treated during the initial SRS session. All systemic and local treatments for BM were recorded. Only two patients in the cohort received bevacizumab as systemic therapy; one experienced intracranial progression, and neither developed RN.

**Table 1:** Patient characteristics

	Total cohort	Asymptomatic RN	Symptomatic RN	Local recurrence
	*n* = 431 (100%)	*n* = 40 (9.3%)	*n* = 36 (8.4%)	*n* = 67 (15.5%)
Age, years				
Mean (SD)	67.0 (9.41)	64.8 (8.8)	62.8 (9.1)	66.9 (8.9)
Median (min-max)	68.4 (30.9-92.7)	66 (44-85)	64.5 (41-79)	69 (40-83)
Histology, *n* (%)				
Adenocarcinoma	322 (74.7)	33 (82.5)	28 (77.8)	51 (76)
SCC	42 (9.7)	4 (10)	3 (8.3)	6 (8.9)
LCLC	13 (3)	0 (0)	0 (0)	1 (1.5)
NOS	24 (5.6)	2 (5)	3 (8.3)	4 (5.9)
Adenosquamous	5 (1.2)	0 (0)	0 (0)	1 (1.5)
SCLC	21 (4.9)	1 (2.5)	2 (5.5)	4 (5.9)
Genetic alterations, *n* (%)				
No alteration	293 (68)	20 (50)	23 (63.9)	41 (61.2)
*EGFR*	39 (9)	6 (15)	5 (13.9)	8 (11.9)
*ALK*	23 (5.3)	6 (15)	3 (8.3)	3 (4.5)
*KRAS*	45 (10.4)	7 (17.5)	4 (11.1)	9 (13.4)
Other	15 (3.5)	1 (2.5)	1 (2.8)	3 (4.5)
Missing	16 (3.7)	0 (0)	0 (0)	2 (2.9)
Number of BM at BM diagnosis, *n* (%)				
1	140 (32.5)	21 (52.5)	11 (30.6)	21 (31.3)
2-3	144 (33.4)	10 (25)	17 (47.2)	21 (31.3)
≥4	147 (34.1)	9 (22.5)	8 (22.2)	25 (37.3)
Diameter of the largest SRS-treated metastasis (mm)				
Mean (SD)	17.7 (7.78)	18.46 (8)	18.90 (7.8)	20 (8.4)
Median (min-max)	18 (2-43)	18 (5-35)	18 (5-31)	20 (4-22.6)
Missing, *n* (%)	4 (0.9)	1 (2.5)	1 (2.7)	0 (0)
Total BM volume during the initial SRS (cm^3^)				
Mean (SD)	4.36 (4.91)	4.3 (3.9)	5.2 (5)	5.6 (5.8)
Median (min-max)	2.81 (0.015-38.2)	3.2 (0.137-15.451)	3.5 (0.16-19.8)	4.2 (0.04-32.53)
Volume of the largest SRS-treated metastasis (cm^3^)				
Mean (SD)	3.69 (4.25)	3.8 (3)	4.5 (4.6)	4.9 (5.7)
Median (min-max)	2.37 (0.0066-32.5)	2.41 (0.1-14.7)	3.3 (0.069-19.8)	3.2 (0.033-32.53)
Time from SRS to RN or LR (months)				
Mean (SD)	—	10.3 (10.4)	8.4 (9.6)	8.9 (7.6)
Median (min-max)	—	6 (2-56)	6 (0.5-57)	6 (1-32)
Histopathological confirmation of RN or LR, *n* (%)				
Yes	—	2 (5)	2 (5.6)	16 (23.9)

BM, brain metastases; LCLC, large-cell lung cancer; LR, local recurrence; NOS, not otherwise specified; RN, radionecrosis; SCC, squamous-cell lung cancer; SCLC, small-cell lung cancer; SD, standard deviation; SRS, stereotactic radiosurgery.

### Impact of tumor volume on the risk of RN and LR

The risks of developing RN, both symptomatic and asymptomatic, as well as symptomatic RN alone, at 6 months and 12 months were evaluated in relation to the total irradiated tumor volume.

**Figure 2:**
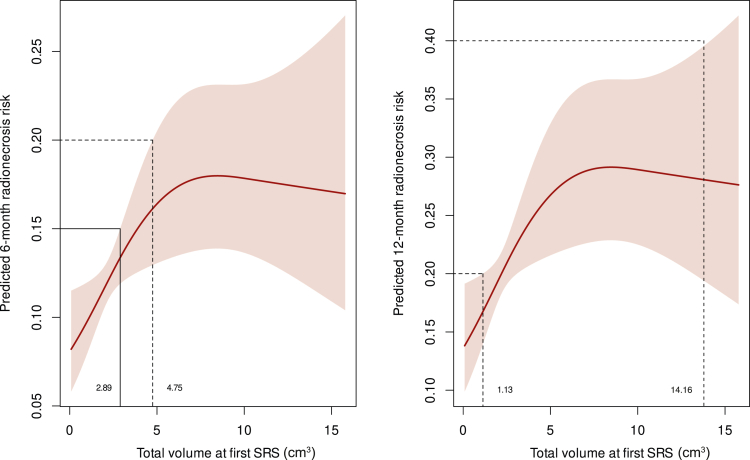
**Predicted 6- and 12-month risk of radionecrosis (RN; both symptomatic and asymptomatic) in relation to total volume of brain metastases.** The left panel shows the predicted 6-month RN risk, while the right panel illustrates the 12-month RN risk. The shaded areas represent 95% confidence intervals. Tumor volumes above certain thresholds are associated with progressively higher risks of RN. For example, at 6 months, tumor volumes exceeding ∼5 cm^3^ are compatible with a risk exceeding 20%. Similarly, at 12 months, tumor volumes as low as 1-2 cm^3^ correspond to a risk exceeding 20%. These relationships highlight the increasing risk of RN with larger treated volumes over time. SRS, stereotactic radiosurgery.

**Figure 3:**
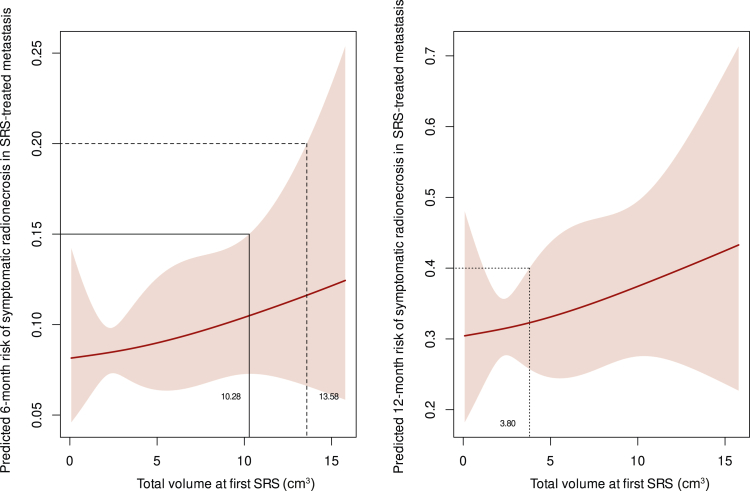
**Predicted 6- and 12-month risk of symptomatic radionecrosis (RN) in relation to total volume of brain metastasis.** The left panel illustrates the predicted 6-month risk of symptomatic RN, while the right panel shows the corresponding 12-month risk. The shaded regions indicate 95% confidence intervals, demonstrating increasing risks of symptomatic RN with larger tumor volumes over time. SRS, stereotactic radiosurgery.

**Figure 4:**
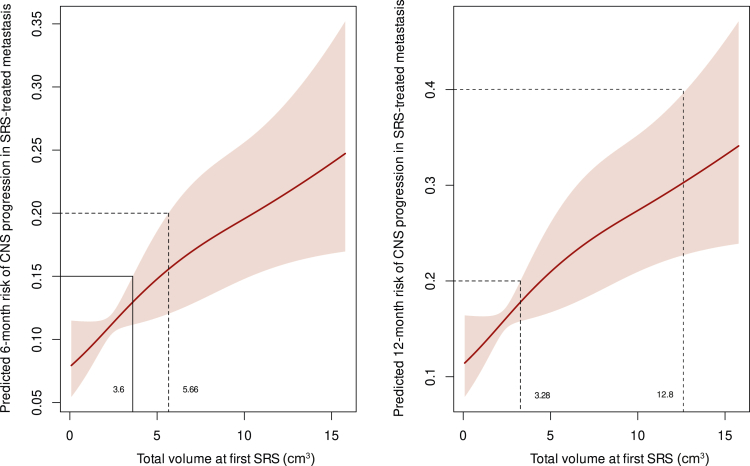
**Predicted 6- and 12-month risk of local recurrence (LR) in relation to total volume of brain metastasis (BM).** The left panel illustrates the predicted 6-month risk of LR, while the right panel shows the corresponding 12-month risk. The shaded regions represent 95% confidence intervals, highlighting the increasing likelihood of LR as the total treated BM volume rises. These visualizations reveal the progressive relationship between tumor volume and recurrence risk. CNS, central nervous system; SRS, stereotactic radiosurgery.

As illustrated in Figure 2, at 6 months, a tumor volume of 4.75 cm^3^ was associated with a 20% risk of RN (estimated risk 16%, 95% CI 13% to 20%), including both symptomatic and asymptomatic cases. By 12 months, significantly smaller volumes, such as 1.13 cm^3^, were sufficient to reach the same RN risk levels (17%, 95% CI 14% to 20%). Symptomatic RN followed a similar trend (Figure 3), with a volume of 13.58 cm^3^ correlating to a 20% risk at 6 months (12%, 95% CI 7% to 20%). By 12 months, much smaller volumes, such as 3.8 cm^3^, were associated with a symptomatic RN risk of 40% (32%, 95% CI 26% to 40%). Findings regarding LR are presented in Figure 4, where a tumor volume of 5.66 cm^3^ was correlated with a 6-month risk of 20% (16%, 95% CI 12% to 20%). At 12 months, smaller volumes (3.28 cm^3^) showed a 20% LR risk (18%, 95% CI 16% to 20%).

**Supplementary Figure 1:**
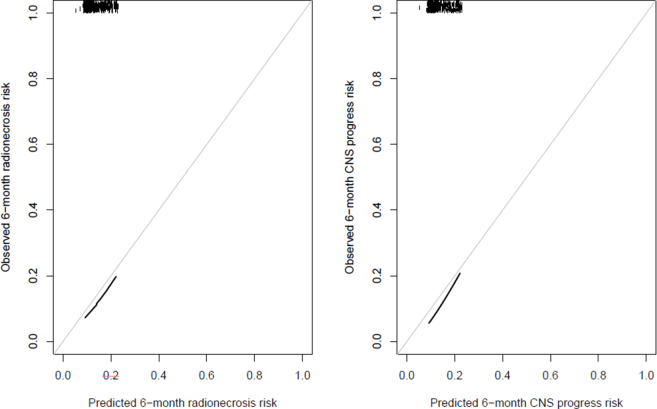


Calibration curves for the prediction of RN and CNS progression indicated strong alignment with optimal predictions, though the ranges of predicted risks were relatively narrow (Supplementary Figure S1, available at https://doi.org/10.1016/j.esmoop.2025.106028).

These findings highlight a significant association between total irradiated tumor volume and the risks of RN and LR. Notably, larger irradiated tumor volumes are particularly related to an increased risk of symptomatic RN, which becomes more pronounced over time.

### Follow-up imaging for risk assessment

Among the 431 patients in the cohort, 15 had missing data on imaging follow-up and were excluded from analysis (assessable *n* = 416). Follow-up imaging was carried out using CT, MRI, or methionine PET-CT. Of the assessable patients, 35 (8.4%) underwent follow-up with CT alone. MRI was utilized for 276 (66.3%) patients, including 126 who received standard contrast-enhanced MRI and 150 who underwent perfusion MRI. Of the remaining 140 patients who did not undergo MRI follow-up, the predominant reason was early death within 4 months of SRS (*n* = 106). Additional causes included rapid clinical deterioration or extensive intracranial progression precluding further SRS, MRI contraindications (*n* = 1, pacemaker), and declined MRI/follow-up (*n* = 2). In this subgroup, follow-up imaging, when available, was most commonly limited to CT carried out for palliative purposes. Methionine PET-CT was carried out in 27 patients with suspected lesion progression or RN; all had prior MRI, and most (*n* = 16) had perfusion MRI. As expected, logistic regression analysis demonstrated that CT alone was less effective than MRI or MRI combined with methionine PET-CT in detecting RN or LR.

### Diagnostic ascertainment of RN and LR

Classification of RN and LR was based primarily on MRI. Methionine PET-CT was reserved for MRI-inconclusive cases, and histopathological confirmation was obtained when imaging remained uncertain or surgery was clinically indicated. Among the 76 RN events, 72 were diagnosed radiologically by MRI (47 perfusion MRI; 25 conventional MRI), and 4 were confirmed histologically. Of the 67 LR cases, 51 were diagnosed by MRI (34 perfusion MRI; 17 conventional MRI), 1 by methionine PET-CT, and 16 by histology (Table 1).

### Diagnostic accuracy of methionine PET-CT

The clinical and demographic characteristics of the 27 patients who underwent methionine PET-CT are summarized in Supplementary Table S2, available at https://doi.org/10.1016/j.esmoop.2025.106028. This subgroup consisted of patients with suspected LR or RN, for whom advanced imaging modalities were required to achieve diagnostic clarification.

The sensitivity and specificity of methionine PET-CT were 0.909 and 0.6, respectively, when MRI was considered the gold standard (Table 2). These results indicate that methionine PET-CT, utilizing an LNR cut-off value >2 (as implemented at KUH), demonstrates high sensitivity for the detection of RN in a real-world clinical setting (Supplementary Figure S2, available at https://doi.org/10.1016/j.esmoop.2025.106028). However, given its modest specificity, methionine PET-CT did not provide a clear advantage over MRI in differentiating LR from RN.

**Table 2:** Diagnostic performance of methionine PET-CT versus MRI for RN and LR

	MRI indicating LR	MRI indicating RN
MET-PET indicating LR	3	2
MET-PET indicating RN	2	20
Statistics	Accuracy: 0.8519 95% CI: 0.6627-0.9581 NIR: 0.8148 *P* value (Acc > NIR): 0.4223 Kappa: 0.5091 McNemar’s test *P* value: 1.0 Sensitivity: 0.9091 Specificity: 0.6 PPV: 0.9091 NPV: 0.6	

Acc, accuracy; CI, confidence interval; LC, local recurrence; MET-PET, methionine positron emission tomography–computed tomography; MRI, magnetic resonance imaging; NIR, no information rate; NPV, negative predictive value; PPV, positive predictive value; RN, radionecrosis.

## Discussion

This study highlights the significance of total irradiated tumor volume in predicting RN and LR in lung cancer patients with CNS involvement treated with SRS. By focusing exclusively on lung cancer, a population with distinct biological and clinical characteristics, our findings provide valuable insights to inform BM management. Additionally, we evaluated the diagnostic performance of methionine PET-CT in differentiating RN from LR in selected cases.

Our data demonstrate a clear association between increased irradiated tumor volume and higher risks of RN and LR, with larger volumes posing a significantly higher risk of symptomatic RN. These findings align with previous studies that have identified tumor size and cumulative volume as key determinants of post-SRS outcomes, although those cohorts included patients with heterogeneous primary malignancies.^15^^,^^24^^,^^29^^,^^30^^,^^37^ Current guidelines discourage SRS for volumes >15 cm^3^, yet our results indicate that clinically relevant risks may emerge even at lower volumes.^1^^,^^37^

The elevated risk of symptomatic RN with larger irradiated volumes is likely multifactorial, encompassing higher radiation doses, tumor necrosis, and more intense inflammatory responses.^30^^,^^38^ In our cohort, the number of BM at diagnosis was evenly distributed between single, two or three, and four or more lesions. Since our analysis focused on total irradiated volume, rather than lesion count, it was difficult to explore whether a single large metastasis confers different risks compared with multiple smaller lesions. Nonetheless, our data suggest that cumulative volume is the dominant driver of both RN and LR risk. The inclusion of patients with a broad spectrum of tumor volumes, including 15 patients with volumes exceeding conventional thresholds, allowed us to delineate volume–risk relationships with greater precision compared with prior studies. This finding holds implications for clinical practice, particularly as systemic therapies extend survival, increasing the likelihood of BM progression or complications.^6^^,^^8^

Local failure followed a similar trend to RN, although the differences in risk between 6 months and 12 months were less pronounced. The association between tumor volume and LR that was documented corroborates prior reports that larger tumor sizes are prone to suboptimal local control.^24^^,^^29^^,^^37^ Although SRS delivers ablative radiation doses, larger tumors may harbor hypoxic regions or subclonal populations resistant to radiotherapy, contributing to recurrence risks.^39^ Our findings emphasize the need for vigilant follow-up, particularly in cases with extensive BM burden.

A major strength of this study is the long follow-up period, allowing for a more comprehensive assessment of RN and LR over time. The selection of 6- and 12-month landmarks for our risk models was guided by the distribution of follow-up in this cohort. The median follow-up was 8 months (IQR 3-19 months), indicating that half of the patients had <1 year of observation. Accordingly, 6 months and 12 months provided clinically meaningful and statistically robust cut points to estimate risk across most of the cohort. Of note, 25 patients were followed for >5 years and 6 for >10 years. This extended observation window enabled us to capture late recurrences and toxicities that might otherwise be underestimated in studies with shorter follow-up. Given the poor prognosis of lung cancer with CNS involvement, the vast majority of patients had deceased by the time of analysis, ensuring a more complete dataset for outcome evaluation.

Differentiating RN from LR remains a persistent challenge in the post-SRS setting. Both conditions can manifest as progressive radiographic abnormalities, complicating clinical decision making.^32^^,^^40^ Some data are encouraging regarding the value of functional imaging modalities, particularly in cases with inconclusive conventional imaging.^35^ In this cohort, methionine PET demonstrated high sensitivity (90.9%) but modest specificity, limiting its added value over MRI. The LNR threshold of >2 applied here has been validated in prior studies; however, variability across institutions underscores the need for standardized protocols to ensure reproducibility.^35^

The clinical implications of our findings span therapeutic strategy, surveillance, and counseling. The observed risks of RN and LR highlight the importance of individualized SRS treatment planning, particularly for patients with large BM volumes. Advances in hypofractionated stereotactic radiotherapy (HF-SRT) offer a potential alternative for such cases, enabling dose de-escalation while preserving tumor control.^29^^,^^41^ Accordingly, total irradiated volume should inform SRS fractionation and stricter normal-brain dose constraints. Volume-based risk can also structure follow-up by prompting closer MRI surveillance for higher risk patients. In counseling, individualized RN/LR risk estimates can be incorporated into shared decision making and consent. For patients with borderline ECOG-PS and high cumulative BM volume, a de-escalation strategy—including deferring SRS to avoid toxicity—may be appropriate. In this way, cumulative treated volume functions as an actionable, pretreatment predictor rather than a merely descriptive correlate.

For patients with a limited number of BM, SRS is preferred over whole-brain radiotherapy (WBRT) because it preserves neurocognition without compromising OS. Adding WBRT improves intracranial control but not OS and worsens cognitive outcomes.^1^^,^^42^, ^43^, ^44^ When broader intracranial control is prioritized, WBRT can be considered.^42^^,^^45^ RN rates are relatively low with WBRT and higher with SRS, reported up to 20% with single-fraction SRS and up to 8% with fractionated SRS.^42^ As RN risk rises with larger BM (e.g. >8 cm^3^), hypofractionated SRS may help mitigate this risk.^42^^,^^46^ Against this backdrop, our results refine SRS decision making by quantifying volume-dependent risks of RN.

Systemic therapy may further influence intracranial outcomes and toxicity. Integrating ICI with SRS shows signals of potential synergistic effects on intracranial control, but concerns remain about increased RN risk, especially in patients with larger lesions.^8^^,^^11^, ^12^, ^13^, ^14^, ^15^^,^^47^, ^48^, ^49^, ^50^ Our study was not designed to evaluate treatment-specific interactions, but these findings highlight the need for future work to clarify how systemic agents influence volume-dependent risks.

In this context, vascular endothelial growth factor (VEGF) inhibitors can induce radiographic shrinkage in both RN and LR, potentially acting as a confounder.^51^, ^52^, ^53^, ^54^ In our cohort, only two patients received bevacizumab as part of systemic therapy; one experienced intracranial progression, while the other had never had any radiological or clinical suspicion of RN or LR. Therefore, bevacizumab exposure could not have confounded the classification of RN versus LR in this study.

The primary limitation of this study lies in its retrospective nature, which inherently presents challenges in the accurate retrieval of symptom and toxicity data from the EMR. Owing to the retrospective design and the absence of standardized volumetric reporting in radiology records, we classified RN as a binary outcome and could not analyze necrosis extent. Although we separately reported symptomatic RN as a proxy for clinical severity, future prospective studies with standardized segmentation (and potentially radiomics) are needed to link necrosis burden and location to symptomatology and outcomes. Our research group is conducting an ongoing radiomics-based trial at Karolinska University Hospital.

Confounding is also possible, as various patient-, tumor-, and treatment-related factors may have influenced results. To mitigate this, we focused exclusively on BM from lung cancer patients, acknowledging the distinct natural histories of different primary diseases. The study was also characterized by variability in the type, timing, combination, and sequencing of systemic therapies, including chemotherapy, ICI, and targeted treatments, administered before and after the diagnosis of BM, as well as in the total number of SRS applications. This heterogeneity posed challenges in precisely analyzing the impact of these interventions. Given the study period from 2009-2020, changes in clinical practice and the introduction of novel therapies over this extended timeframe may have influenced treatment decisions and outcomes. This study was conducted at a single center, which may limit its generalizability. However, the inclusion of all-comers in a real-world cohort enhances the clinical relevance of our results.

Furthermore, variability in imaging interpretation may have influenced diagnostic accuracy, which depends on radiological expertise and differences in imaging modalities across patients. In our study, the subgroup of patients who underwent methionine PET-CT was highly selected, reflecting real-world practice. Methionine PET was pursued only when MRI findings remained inconclusive, typically in cases with larger or diagnostically challenging lesions. This selection may have underestimated the potential added value of PET over MRI in a broader, unselected population. Only a small proportion of cases had histopathological confirmation (*n* = 20; 4 RN and 16 LR), reflecting routine clinical practice in which surgical sampling is reserved for diagnostically or therapeutically compelling scenarios. Moreover, while perfusion MRI provides superior accuracy compared with conventional MRI, false positives still occur, underscoring the continued difficulty of reliably distinguishing RN from LR in clinical practice. These limitations are consistent with prior studies showing that even perfusion MRI cannot reliably differentiate RN from recurrent metastases, with histopathology remaining the diagnostic gold standard.^55^

Another limitation is the use of CT during follow-up for a small proportion of our patients due to early death or rapid clinical decline, which may have constrained the accuracy of diagnosing asymptomatic RN. While this reduces overall diagnostic accuracy, potential errors in distinguishing RN from LR are likely random and infrequent across the cohort, representing a form of nondifferential misclassification. Furthermore, because many of these patients had very short survival, any undetected RN or LR cases would likely have led to underestimation rather than overestimation of event rates. The extended follow-up period and consistent diagnostic methodology used in this study minimize the overall risk of misclassification. Incorporating more advanced imaging modalities in routine clinical follow-up could improve diagnostic precision and warrants consideration in future studies. Finally, the findings presented in this analysis require external validation to ensure their generalizability and robustness across different patient populations and clinical settings.

Future research should prioritize the external and prospective validation of our volume–risk models, with a focus on leveraging advanced imaging techniques, including radiomics. By capturing spatial heterogeneity and phenotypic traits of BM, radiomics offers promising opportunities to enhance risk stratification and treatment personalization through the identification of predictive imaging markers for outcomes or toxicity.^56^^,^^57^ In parallel, integration of systemic therapy data, patient-reported outcomes, and neurocognitive metrics into SRS trials will enable a more comprehensive assessment of therapeutic effects and foster the development of individualized care pathways.

### Conclusion

In conclusion, this study highlights the importance of total irradiated tumor volume as a predictor of RN and LR in lung cancer patients undergoing SRS. Our findings reveal that even patients with relatively small or moderate BM volumes remain at risk for RN, particularly those with longer expected survival. While methionine PET-CT demonstrated high sensitivity in detecting RN, its limited specificity restricts its role as a definitive diagnostic tool. Future research should validate these models externally and explore the integration of radiomics, novel systemic therapies, and patient-centered outcomes to optimize treatment strategies.

## Figures and Tables

**Supplementary Figure 2:**