# Supplementary material for: Irradiated tumor volume as a predictor of local recurrence and radionecrosis in lung cancer with brain metastases treated with stereotactic radiosurgery
Source: ESMO Open. 2026 Jan 2;11(1):106028. doi: 10.1016/j.esmoop.2025.106028 (PMC12805339; doi:10.1016/j.esmoop.2025.106028)
Supplement: Supplementary Table 1 [file mmc1.docx]

**Supplementary Table S1. Comprehensive list of registered variables**

| VARIABLES |
| --- |
| 1. Gender |
| 1. Date of birth |
| 1. Smoking status   (Non smoker, Ex-smoker, Smoker) |
| 1. Comorbidities   (No, Cardiovascular, Pulmonary, Autoimmune/Immunodeficiency, Other malignancies) |
| 1. Date of lung cancer diagnosis |
| 1. TNM staging at diagnosis |
| 1. Stage at lung cancer diagnosis |
| 1. Surgery for primary tumor |
| 1. Imaging CNS at BM diagnosis   (No imaging, CT, MRI) |
| 1. Biopsy/surgery of BM   (No, Biopsy, Surgery) |
| 1. Date of BM diagnosis |
| 1. Age at diagnosis of BM |
| 1. Age at first SRS |
| 1. Biopsy of primary tumor/ extracranial metastases at the time of primary tumor diagnosis   (No, Primary tumor, Mediastinal lymph nodes, Extrathoracic) |
| 1. Histology primary tumor   (Lung ADC, SCC, LCLC, NOS, SCLC) |
| 1. Molecular status of primary tumor   (No alteration, EGFR, ALK, BRAF, ROS1, KRAS, Other) |
| 1. PD-L1 status primary tumor   (0%, 1-50%, ≥50%, Not available) |
| 1. Extracranial biopsy at BM diagnosis   (No, Primary tumor, Mediastinal lymph nodes, Extrathoracic) |
| 1. BM at diagnosis of primary tumor |
| 1. ECOG-PS at BM diagnosis |
| 1. Extracranial disease at BM diagnosis |
| 1. Disease outside CNS under control |
| 1. GPA Class |
| 1. Lungmol-GPA |
| 1. BSBM |
| 1. SIR |
| 1. Number of BM at BM diagnosis |
| 1. Symptomatic CNS disease |
| 1. Leptomeningeal disease after the first SRS application * |
| 1. First-line treatment for BM   (No treatment, WBRT, Surgery, γ-knife, CΤ, TKI, Immunotherapy, CT plus Immunotherapy) |
| 1. Received treatment (all treatments that have been given) for BM   (No treatment, WBRT, Surgery, γ-knife, CΤ, TKI, Immunotherapy, CT plus Immunotherapy) |
| 1. Received treatment before the diagnosis of BM   (No treatment, WBRT, Surgery, γ-knife, CT, TKI, Immunotherapy, CT plus Immunotherapy) |
| 1. Date of first SRS |
| 1. Size of the largest SRS-treated BM |
| 1. The exact size of the largest SRS-treated BM |
| 1. The total diameter of all SRS-treated BM |
| 1. Number of SRS-treated BM including all SRS applications |
| 1. Number of SRS-treated BM during the first SRS application |
| 1. Radionecrosis after SRS |
| 1. Symptomatic radionecrosis |
| 1. Months after SRS when radionecrosis occurred |
| 1. CNS progress after SRS   (No, Yes in SRS-treated BM, Yes outside SRS-treated BM) |
| 1. Months after SRS when CNS progress occurred |
| 1. Met-PET after SRS |
| 1. MRI after SRS   (No, Without perfusion, With perfusion) |
| 1. CT brain after SRS |
| 1. Met-PET diagnosis correct   (No, Yes verified without surgery, Yes verified with surgery) |
| 1. MRI diagnosis correct   (No, Yes verified without surgery, Yes verified with surgery) |
| 1. CT diagnosis correct   (No, Yes verified without surgery, Yes verified with surgery) |
| 1. Death |
| 1. Death cause   (Cancer-related, Cancer-related due to BM, Other causes of death when cancer disease under control) |
| 1. Date of death |

TNM: Tumour, Node, Metastasis; CNS: Central Nervous System; BM: Brain Metastases; CT: Computed Tomography; MRI: Magnetic Resonance Imaging; SRS: Stereotactic Radiosurgery; ADC: Adenocarcinoma; SCC: Squamous Cell Lung Cancer; LCLC: Large Cell Lung Cancer; NOS: Not Otherwise Specified; SCLC: Small Cell Lung Cancer; PD-L1: Programmed Death-Ligand 1; ECOG-PS: Eastern Cooperative Oncology Group Performance Status; GPA: Graded Prognostic Assessment; BSBM: Basic Score for Brain Metastases; SIR: Score Index for Radiosurgery; WBRT: Whole Brain Radiation Therapy; TKI: Tyrosine Kinase Inhibitors; CT: Chemotherapy; Met-PET: Methionine Positron Emission Tomography.

*Patients who developed leptomeningeal carcinomatosis following the first SRS application during the course of the disease.
