# Supplementary material for: Irradiated tumor volume as a predictor of local recurrence and radionecrosis in lung cancer with brain metastases treated with stereotactic radiosurgery
Source: ESMO Open. 2026 Jan 2;11(1):106028. doi: 10.1016/j.esmoop.2025.106028 (PMC12805339; doi:10.1016/j.esmoop.2025.106028)
Supplement: Supplementary Table 2 [file mmc2.docx]

|  | Patients examined with methionine PET-CT |
| --- | --- |
|  | N=27 |
| Age |  |
| Mean (SD) | 59.9 (9.5) |
| Median [Min, Max] | 60.0 [41-73] |
| Histology |  |
| Adenocarcinoma | 23 (85%) |
| SCC | 2 (7.4%) |
| LCLC | 0 (3%) |
| NOS | 1 (3.7%) |
| Adenosquamous | 0(0%) |
| SCLC | 1 (3.7%) |
| Genetic alterations |  |
| No alteration | 18 (66.0%) |
| *EGFR* | 1(3.7%) |
| *ALK* | 2 (7.4%) |
| *KRAS* | 5 (18.5%) |
| Other | 1 (3.7%) |
| Missing | 0 (0%) |
| Diameter of the largest SRS-treated metastasis (mm) | |
| Mean (SD) | 20.6 (7.7) |
| Median [Min, Max] | 20 [4-35] |
| Missing | 4 (0.9%) |
| Total BM volume during the initial SRS (cm^3^) | |
| Mean (SD) | 5.4 (6.7) |
| Median [Min, Max] | 3.6 [0.006, 32.5] |
| The volume of the largest SRS-treated metastasis (cm^3^) | |
| Mean (SD) | 5.0 (6.4) |
| Median [Min, Max] | 3.0 [0.006, 32.5] |

**Supplementary Table 2. Clinical and demographic characteristics of patients examined with methionine PET-CT**

SD: Standard Deviation; SCC: Squamous Cell Lung Cancer; LCLC: Large Cell Lung Cancer; NOS: Not Otherwise Specified; SCLC: Small Cell Lung Cancer; SRS: Stereotactic Radiosurgery; BM: Brain Metastases
