# Supplementary material for: Irradiated tumor volume as a predictor of local recurrence and radionecrosis in lung cancer with brain metastases treated with stereotactic radiosurgery
Source: ESMO Open. 2026 Jan 2;11(1):106028. doi: 10.1016/j.esmoop.2025.106028 (PMC12805339; doi:10.1016/j.esmoop.2025.106028)
Supplement: Supplementary data [file mmc3.docx]

**Prediction of radionecrosis (RN) and local recurrence (LR)**

Calibration curves for the prediction of radionecrosis and CNS progress can be constructed using the Efron-Gong bootstrap (Supplementary Figure S1). While the calibration curves were close to optimal, the ranges of predicted risks were narrow.

Supplementary Figure S1. Calibration plots of the predicted (horizontal axis) and the observed (vertical axis) risks of radionecrosis (left) and CNS progress (right). The grey 45-degree lines are the lines of perfect calibration where the predicted and the observed risks match. The black bold lines are the optimism-corrected estimates of the calibration. Predicted six-month risks are shown as tick marks at the top of the figures.

**Contrasting MR and Methionine PET-CT**

Supplementary Figure S2. The probability of detecting RN (left panel) or LR (right panel) based on whether an MRI, or both MRI and methionine PET-CT, were performed.
